# RETRACTION: Impact of Open and Minimally Invasive Surgery on Postoperative Wound Complications in Patients Undergoing Prostate Surgery: A Meta‐Analysis

**DOI:** 10.1111/iwj.70341

**Published:** 2025-03-17

## Abstract

**RETRACTION:** GongX. and XuR. , “Impact of Open and Minimally Invasive Surgery on Postoperative Wound Complications in Patients Undergoing Prostate Surgery: A Meta‐Analysis,” International Wound Journal 21, no. 1 (2024): e14367, 10.1111/iwj.14367.37706271 PMC10788585

The above article, published online on 14 September 2023, in Wiley Online Library (http://onlinelibrary.wiley.com/), has been retracted by agreement between the journal Editor in Chief, Professor Keith Harding; and John Wiley & Sons Ltd. Following an investigation by the publisher, all parties have concluded that this article was accepted solely on the basis of a compromised peer review process. The editors have therefore decided to retract the article. The authors did not respond to our notice regarding the retraction.

**RETRACTION:** X. Gong and R. Xu , “Impact of Open and Minimally Invasive Surgery on Postoperative Wound Complications in Patients Undergoing Prostate Surgery: A Meta‐Analysis,” International Wound Journal 21, no. 1 (2024): e14367, 10.1111/iwj.14367.37706271 PMC10788585

The above article, published online on 14 September 2023, in Wiley Online Library (http://onlinelibrary.wiley.com/), has been retracted by agreement between the journal Editor in Chief, Professor Keith Harding; and John Wiley & Sons Ltd. Following an investigation by the publisher, all parties have concluded that this article was accepted solely on the basis of a compromised peer review process. The editors have therefore decided to retract the article. The authors did not respond to our notice regarding the retraction.

